# Case Report: Pulmonary artery biopsy findings in a patient with a BMPR2 variant-associated pulmonary arterial hypertension

**DOI:** 10.3389/fmed.2026.1848607

**Published:** 2026-07-01

**Authors:** Hongxia Wu, Li Guo, Weiya Wang, Faming Jiang, Ye Wang

**Affiliations:** 1 Department of Respiratory and Critical Care Medicine, West China Hospital, Sichuan University, Chengdu, China; 2 Department of Pathology, West China Hospital, Sichuan University, Chengdu, China

**Keywords:** BMPR2, inflammation, lipidomics, pathology, pulmonary arterial hypertension

## Abstract

Mutations in BMPR2 are the most common genetic cause of heritable pulmonary arterial hypertension (PAH). We report a 47-years-old male with a BMPR2 variant (c.246A > G) who presented with dyspnea and recurrent hemoptysis. Right heart catheterization confirmed PAH with a mean PAP of 46 mmHg, PAWP of 3 mmHg, and PVR of 10.75 WU. A pulmonary artery biopsy was performed to exclude alternative diagnoses such as vasculitis and chronic thromboembolism. Histology showed foam cell accumulation in the intima, infiltration of CD3^+^ T cells, CD20^+^ B cells, and CD68^+^ histiocytes in the intima and media, and mucoid deposition. These findings suggest an active inflammatory component and dysregulation of lipid metabolism in early-stage PAH. Targeted PAH therapy improved symptoms, and resolved hemoptysis after bronchial artery embolization. Although the variant is of uncertain significance, this rare biopsy provides early pathological insights into BMPR2-associated PAH. Further studies are needed to clarify mechanisms linking BMPR2, inflammation, and lipid metabolism in vascular remodeling.

## Introduction

Mutations in the bone morphogenetic protein receptor type II (BMPR2) gene represent the most common causal factor for hereditary pulmonary arterial hypertension (PAH) (1). Pulmonary vascular remodeling is a hallmark of all forms of PAH and is characterized by a range of structural and functional changes that primarily affect the distal pulmonary circulation (2, 3). This process predominantly affects the distal pulmonary circulation and involves increased fractional thickness of the intima and media of the pulmonary artery (3). Metabolic alterations and inflammatory mechanisms have also been implicated in the pathogenesis of PAH (3). Notably, PAH arising from different etiologies exhibits distinct pathological features. Studies investigating the morphological alterations and potential underlying causes of PAH have substantially advanced the understanding of this complex disease.

Currently, pathological research on PAH mainly relies on transplanted lungs or autopsy samples. Obtaining early pathological specimens from PAH patients remains challenging. This report describes the pathological features of pulmonary artery biopsy specimens from a case diagnosed with BMPR2 variant-associated PAH.

## Case presentation

A 47-years-old male presented with a 6-years history of dyspnea and recurrent hemoptysis. Right heart catheterization (RHC) performed at a previous institution revealed pulmonary artery hypertension (PAH) and elevated pulmonary vascular resistance (PVR). Although targeted drug therapy (macitentan 10 mg daily, tadalafil 10 mg daily, and sirupag 0.4 mg in the morning and 0.2 mg in the afternoon for 3 months) for PAH improved dyspnea, recurrent hemoptysis persisted, leading to referral for further management.

**FIGURE 1:**
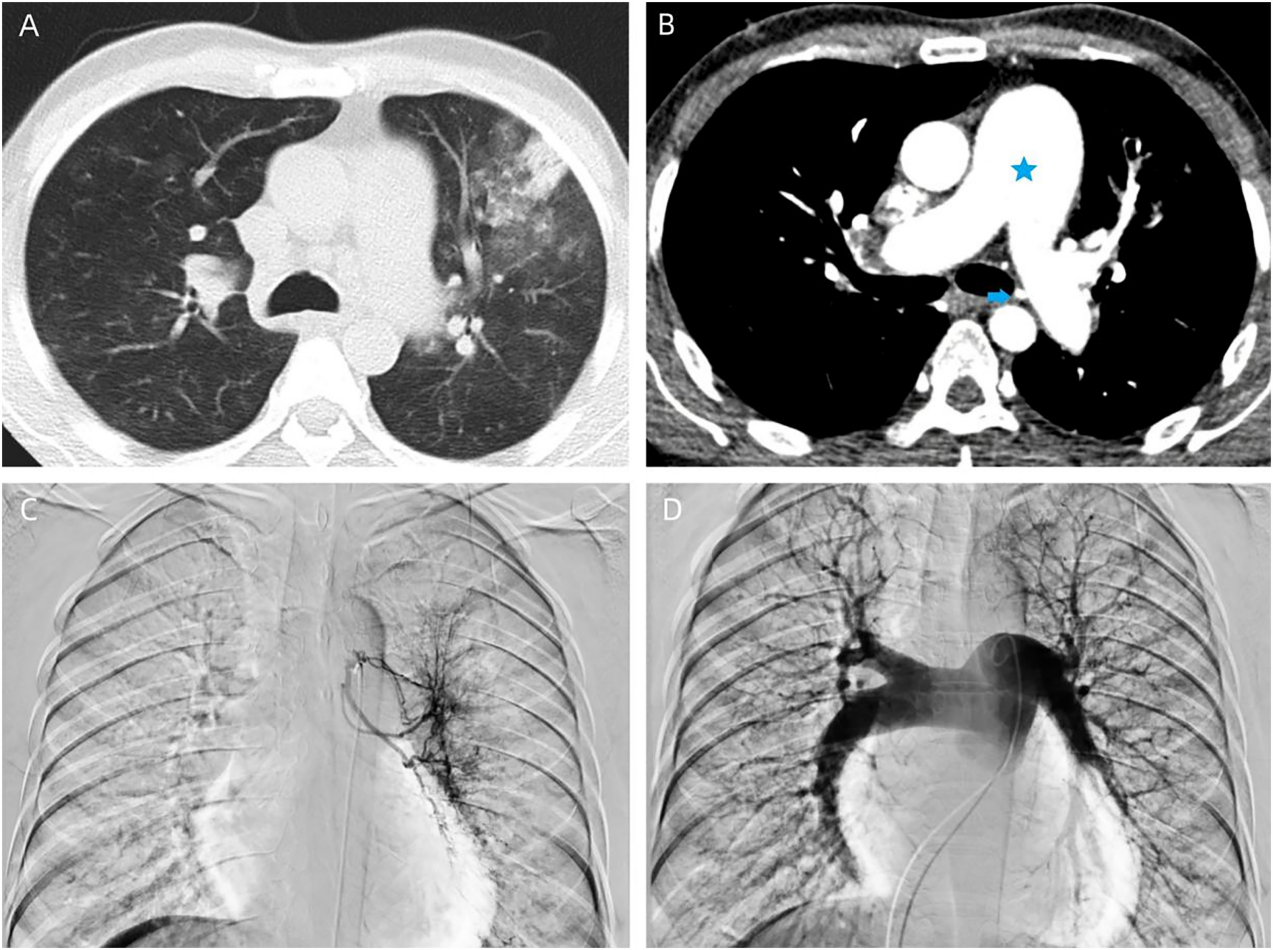
Imagings of the case. **(A)** CT scan shows effusions in the left lung. **(B)** CT angiography reveals dilated pulmonary arteries (star) and tortuous bronchial arteries (arrow). **(C)** Bronchial artery angiography demonstrates dilated and tortuous bronchial arteries. **(D)** Pulmonary artery angiography reveals dilation of the main and bilateral pulmonary arteries, with sparse visualization of distal pulmonary arteries.

Upon admission, physical examination found jugular venous distension, right ventricular heave, and loud P2. Six-minute walk test: 478 meters. World Health Organization (WHO) functional class II. Laboratory evaluation indicated a hemoglobin level of 108 g/L, with normal liver and kidney function, rheumatoid immune indicators. Lipid profiles, including total cholesterol, LDL, HDL, and triglycerides, are within normal limits. Blood gas analysis on room air indicated a pH of 7.396, PO_2_ of 65.2 mmHg, and PCO2 of 38.7 mmHg. The N-terminal pro-brain natriuretic peptide concentration was 553 ng/L. Enhanced CT scans revealed a left lung effusion, a dilated pulmonary artery, and tortuous bronchial arteries (Figures 1A, B), without evidence of atherosclerosis. Echocardiography demonstrated tricuspid valve regurgitation (Vmax = 4.6 m/s) and an estimated pulmonary artery systolic pressure of 113 mmHg.

**FIGURE 2:**
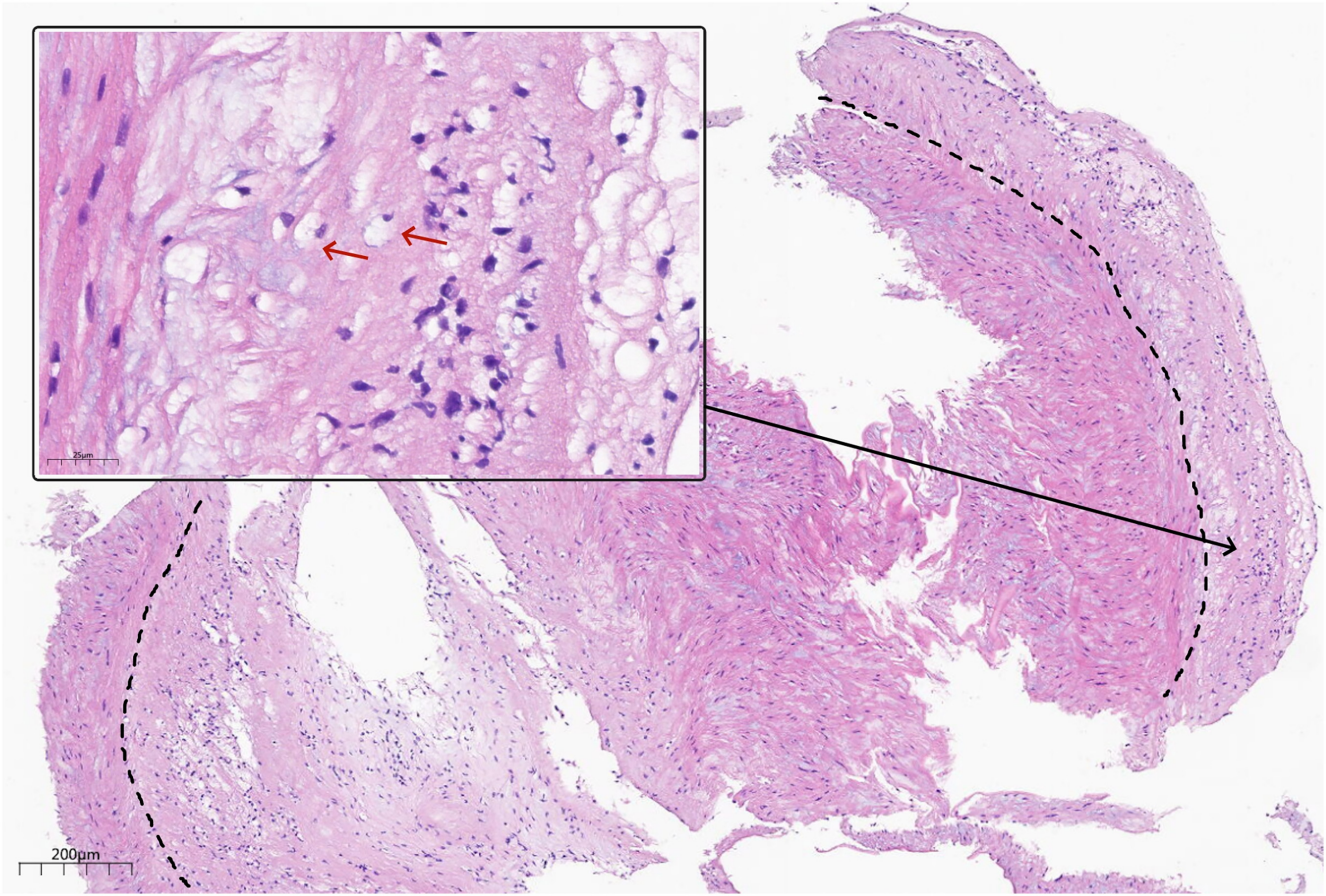
Photomicrographs of staining of pulmonary artery biopsy specimens. Histological morphology of the pulmonary artery biopsy tissue (H&E). Scale label 200 μm; H&E, hematoxylin and eosin; Blue arrows, foam cell; Intima/media boundaries: dotted lines.

**FIGURE 3:**
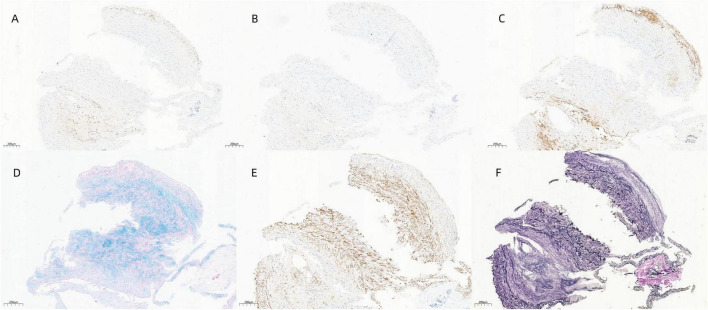
Photomicrographs of staining and immunohistochemistry of pulmonary artery biopsy specimens. Pathology shows the histological morphology of the pulmonary artery biopsy tissue, with a foam cell reaction, B lymphocytes, T lymphocytes and histiocytes infiltration in intima and media, and mucoid deposition between vessel walls. **(A)** Positive CD3 stain, indicating significant T lymphocytes accumulation within the intima and media (scale label 200 μm). **(B)** Positive CD20 stain, indicating significant B lymphocytes accumulation within the intima and media (scale label 200 μm). **(C)** Positive CD68PG-M1 stain, indicating CD68-positive histiocytes infiltration within the intima and media of the pulmonary artery, consistent with foam cell accumulation (scale label 200 μm). **(D)** Positive AB stain, indicating significant mucoid deposition between vessel walls (scale label 200 μm). **(E)** Positive α-SMA stain (scale label 200 μm). **(F)** Positive elastic stain (scale label 200 μm). AB, Alcain blue.

Bronchial artery angiography demonstrated tortuous bronchial arteries with marked distal contrast agent staining (Figure 1C). Bronchial artery embolization was performed to control recurrent hemoptysis. Pulmonary artery angiography revealed dilation of the main and bilateral pulmonary arteries, with limited visualization of distal branches (Figure 1D). RHC revealed a pulmonary artery pressure of 88/24 (46) mmHg, a pulmonary artery wedge pressure of 3 mmHg, a PVR of 10.75 Wood units, a cardiac output of 4.0 L/min, a cardiac index of 2.6 L/min/m^2^, and a right atrial pressure of 2 mmHg. A pulmonary artery biopsy was performed to assess underlying pathological changes after multidisciplinary discussion. The biopsy was performed due to unexplained hemoptysis and suspicion of a localized vascular abnormality based on CT imaging and angiography, such as vasculitis or chronic pulmonary embolism, which could not be confirmed by current non-invasive methods. A bronchial blocker was placed at the biopsy site during the procedure to prevent bleeding. Three tissue samples were obtained from the basal segment of the left lower lobe using myocardial biopsy forceps with airway protection. No adverse reactions related to the biopsy were observed. The samples were yellowish-white and measured 0.1–0.3 cm in diameter. Histological examination revealed a pronounced foam cell accumulation in the intima, infiltration of B lymphocytes, T lymphocytes, and histiocytes in both the intima and media, and mucoid deposition between vessel walls (Figures 2, 3, Supplementary Figure 1). No plexiform lesion, intimal fibrosis, or thrombosis was observed. Assessments of cellular proliferation and vascular occlusion were limited due to incomplete collection of pulmonary artery tissue. A BMPR2 gene variant (NM_001204.7:c.246A > G(p.Gln82=)) was identified; however, family genetic analysis was not performed because the patient’s parents are deceased. At 3 and 6 months of follow-up, the patient continued PAH targeted drug therapy without adverse events, experienced improved dyspnea (WHO functional class from II to I), and resolved hemoptysis.

## Discussion

The diagnosis of PAH is typically confirmed through RHC, echocardiography, and exclusion of other causes. Invasive pulmonary artery biopsy is not standard practice for clinically established PAH. Biopsy carries significant risks (hemoptysis, pneumothorax) and rarely alters management in idiopathic/heritable PAH. In this case, the biopsy was clinically justified to rule out alternative diagnoses, and we now explicitly state that the biopsy results did not change the overall management of PAH but provided valuable pathological insights.

Pulmonary artery pathology associated with lipid metabolism and inflammation disorders is infrequently reported, especially in the context of BMPR2 variant-associated PAH. Whether inflammatory cell infiltration and lipid metabolism disorders represent common early pathological features of PAH or are unique to BMPR2 variant-associated PAH remains undetermined. However, early collection of specimens in PAH may facilitate timely guidance and evaluation of therapeutic effects.

The histological findings of infiltration by CD3^+^ T lymphocytes, CD20^+^ B lymphocytes, and CD68^+^ histiocytes indicate that the pulmonary vascular lesion is not merely a structural defect but an active inflammatory disease. BMPR2 deficiency contributes to PAH pathogenesis by interacting with genetic mutations, inflammation, and endothelial dysfunction, leading to pathological vascular remodeling (4, 5). BMPR2 insufficiency is linked to altered vascular cell behaviors relevant to pulmonary vascular remodeling, including changes in proliferation, survival, and differentiation (6). Pulmonary artery endothelial cells show impaired homeostasis in the absence of BMPR2. This includes increased apoptosis, defective repair, and apoptosis-resistant, hyperproliferative subpopulations (7). These changes likely contribute to characteristic PAH lesions such as arterial wall thickening and complex vascular remodeling (8, 9). Kit-Yee Chu reported a receptor–dosage model in which physiological BMPR2 expression is required to sustain homeostatic BMP9/10 signaling in pulmonary artery endothelium (10). A significant increase in perivascular inflammatory cell infiltration, including macrophages, macrophages/monocytes, mast cells, dendritic cells, T cells, was found in pulmonary vascular lesions of patients with idiopathic PAH (11). In this context, inflammatory cell infiltration may be considered a primary pathogenic participant, as BMPR2 insufficiency transforms pulmonary artery endothelial cells into an inflammatory platform that retains and activates macrophages and T cells. Although there is significant infiltration of inflammatory cells in the pulmonary arteries of PAH patients and vascular remodeling occurs, it is not yet clear whether this relates to the patients’ hemoptysis.

The formation of macrophage-derived foam cells represents a critical connection to atherosclerotic lesions, vascular injury, and chronic inflammation. Bordag reported that explanted lungs from patients with idiopathic PAH exhibit lipid accumulation in both the intima and media, as well as altered expression of genes related to lipid homeostasis (12). Bone morphogenetic protein (BMP) modulates macrophage function, while macrophages reciprocally regulate the BMP signaling system (1). Reduced expression of BMPR2 increases granulocyte-macrophage colony-stimulating factor translation and promotes macrophage recruitment in both humans and mice, thereby worsening PAH (13). The endothelial CD74–macrophage migration inhibitory factor axis further facilitates the recruitment of macrophages and lymphocytes. In animal models, impaired lipid metabolism is the primary mechanism responsible for lipid deposition associated with BMPR2 mutation, and dietary lipid intake further intensifies this effect (14).

Strength of this study includes the rare availability of biopsy tissue in early PAH. Limitations include the single case, lack of functional studies for the variant, absence of lipid-specific staining, and speculative nature of the association. The variant of BMPR2 is of uncertain significance and that the case is presented as an association, not a proven causal link. The association with lipid metabolism is now presented as a hypothesis, not as a central claim. We recommend cautious interpretation.

This rare biopsy in BMPR2-variant PAH reveals foam cells, T/B-cell infiltration, and mucoid deposition, suggesting inflammation and lipid dysregulation may contribute to early vascular remodeling. Further investigations are needed to clarify the mechanistic links among BMPR2 insufficiency, immune activation, and metabolic alterations, which may inform targeted therapeutic strategies.

Patient perspective: the biopsy was scary, but I am glad it helped rule out other diseases.

## Data Availability

The original contributions presented in this study are included in the article/Supplementary material, further inquiries can be directed to the corresponding authors.

## Supplementary material

Supplementary Figure 1 CD31 staining of pulmonary artery biopsy specimens.
