# Supplementary figures and images for: Case Report: Pulmonary artery biopsy findings in a patient with a BMPR2 variant-associated pulmonary arterial hypertension
Source: Front Med (Lausanne). 2026 Jul 1;13:1848607. doi: 10.3389/fmed.2026.1848607 (PMC13368745; doi:10.3389/fmed.2026.1848607)

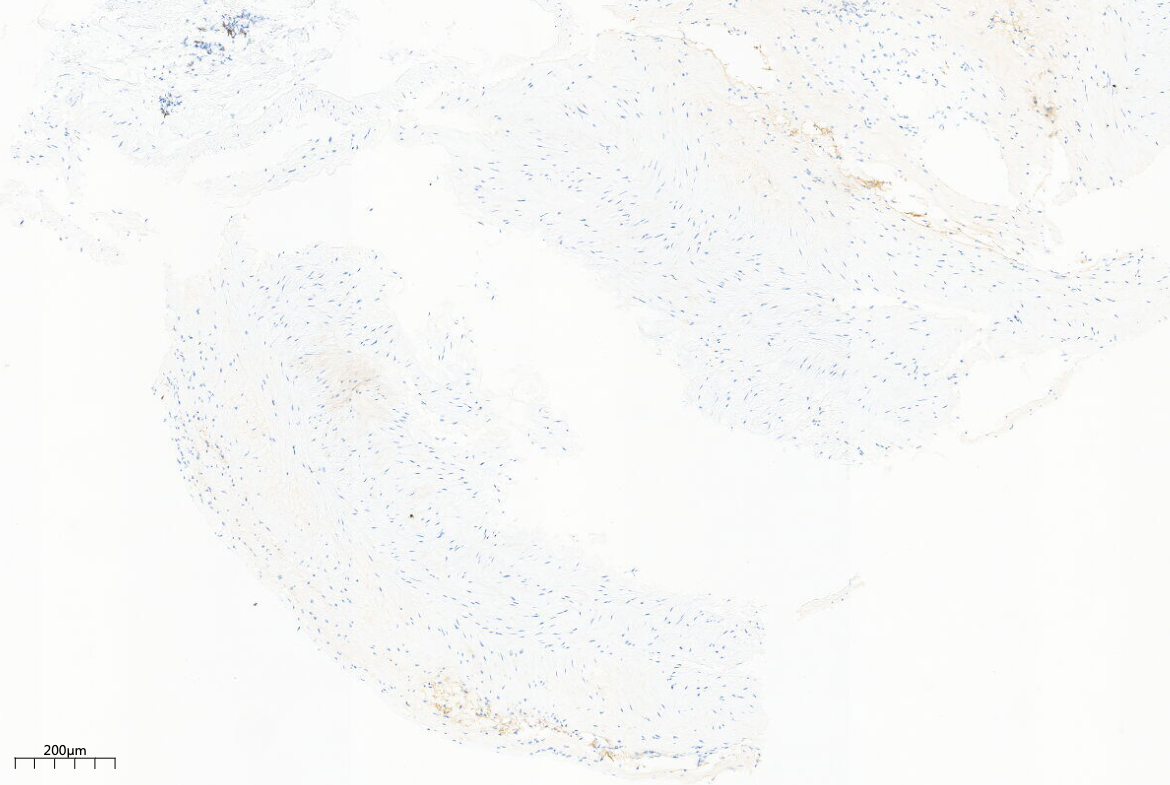

Supplement: Supplementary Figure 1 — CD31 staining of pulmonary artery biopsy specimens. [file Image_1.TIF]
